# Impact of the Addition of a Centrifugal Pump in a Preterm Miniature Pig Model of the Artificial Placenta

**DOI:** 10.3389/fphys.2022.925772

**Published:** 2022-07-22

**Authors:** Alex J. Charest-Pekeski, Steven K. S. Cho, Tanroop Aujla, Liqun Sun, Alejandro A. Floh, Mark J. McVey, Ayman Sheta, Marvin Estrada, Lynn Crawford-Lean, Celeste Foreman, Dariusz Mroczek, Jaques Belik, Brahmdeep S. Saini, Jessie Mei Lim, Olivia J. Moir, Fu-Tsuen Lee, Megan Quinn, Jack R. T. Darby, Mike Seed, Janna L. Morrison, Christoph Haller

**Affiliations:** ^1^ Department of Physiology, Temerty Faculty of Medicine, University of Toronto, Toronto, ON, Canada; ^2^ Translational Medicine, The Hospital for Sick Children, Toronto, ON, Canada; ^3^ Early Origins of Adult Health Research Group, Health and Biomedical Innovation, UniSA: Clinical and Health Sciences, University of South Australia, Adelaide, SA, Australia; ^4^ Division of Cardiology, The Labatt Family Heart Centre, The Hospital for Sick Children, Toronto, ON, Canada; ^5^ Department of Critical Care Medicine, The Hospital for Sick Children, Toronto, ON, Canada; ^6^ Department of Anesthesiology and Pain Medicine, The Hospital for Sick Children, University of Toronto, Toronto, ON, Canada; ^7^ Department of Physics, Ryerson University, Toronto, ON, Canada; ^8^ Department of Pediatrics, Division of Neonatology, The Hospital for Sick Children, Toronto, ON, Canada; ^9^ Lab Animal Services, Peter Gilgan Center for Research and Learning, The Hospital for Sick Children, Toronto, ON, Canada; ^10^ Division of Cardiovascular Surgery, The Labatt Family Heart Centre, The Hospital for Sick Children, University of Toronto, Toronto, ON, Canada; ^11^ Institute of Medical Science, Temerty Faculty of Medicine, University of Toronto, Toronto, ON, Canada

**Keywords:** artificial placenta, preterm pig, cannulation, centrifugal pump, oxygenator, tachycardia, fetal development

## Abstract

The recent demonstration of normal development of preterm sheep in an artificial extrauterine environment has renewed interest in artificial placenta (AP) systems as a potential treatment strategy for extremely preterm human infants. However, the feasibility of translating this technology to the human preterm infant remains unknown. Here we report the support of 13 preterm fetal pigs delivered at 102 ± 4 days (d) gestation, weighing 616 ± 139 g with a circuit consisting of an oxygenator and a centrifugal pump, comparing these results with our previously reported pumpless circuit (*n* = 12; 98 ± 4 days; 743 ± 350 g). The umbilical vessels were cannulated, and fetuses were supported for 46.4 ± 46.8 h using the pumped AP versus 11 ± 13 h on the pumpless AP circuit. Upon initiation of AP support on the pumped system, we observed supraphysiologic circuit flows, tachycardia, and hypertension, while animals maintained on a pumpless AP circuit exhibited subphysiologic flows. On the pumped AP circuit, there was a progressive decline in umbilical vein (UV) flow and oxygen delivery. We conclude that the addition of a centrifugal pump to the AP circuit improves survival of preterm pigs by augmenting UV flow through the reduction of right ventricular afterload. However, we continued to observe the development of heart failure within a matter of days.

## Introduction

In resource rich nations, extreme prematurity, defined as delivery prior to 28 weeks’ gestation, remains the leading cause of childhood mortality and morbidity (Matthews et al., 2015; Patel et al., 2015). Survival rates decline with decreasing gestational age (GA), with only 6% surviving at 22 weeks GA compared to >90% surviving at 28 weeks GA (Stoll et al., 2010). In Canada alone, the economic burden of caring for extremely preterm infants approaches $600 million nationally per annum (Blencowe et al., 2013). Unfortunately, despite advances in medical technology and neonatal intensive care, improvements in the outcomes of children born extremely preterm over the last 15 years have been limited (Lee et al., 2020).

At the biological limit of viability (22–25 weeks GA), preterm infants commence pulmonary gas exchange during the late-canalicular and early saccular stages of lung development. Exposure to the positive pressure mechanical ventilation and high partial pressure of inspired oxygen required to achieve adequate gas exchange results in cessation of alveolarization and pulmonary microvascular injury, which is associated with high rates of chronic lung disease and pulmonary hypertension (Thébaud et al., 2019). Furthermore, approximately 50% of children born at the threshold of viability exhibit neurological disabilities at 30 months corrected age, with half of these cases being classified as severe (Wood et al., 2000). Despite major advances in the outcomes of prenatal infants resulting from the widespread administration of prenatal steroids and exogenous surfactant, recent improvements in the morbidity and mortality have been more incremental, emphasizing the need for new innovative approaches to supporting the fragile physiology and development of extremely preterm infants.

An artificial placenta (AP) represents a novel approach that aims to maintain the innate fetal circulation while promoting normal prenatal development. Gas exchange is achieved with a low-resistance hollow-fiber membrane oxygenator connected to the fetus *via* the umbilical vasculature, while incubating the fetus in a fluid filled environment. The first attempt to support previable human infants was reported in the late 1950s (Westin et al., 1958); with subsequent progress made using animal models. These early experiments were complicated by the development of heart failure and infections and concurrent improvements in conventional neonatal intensive care led to diminished enthusiasm for AP technology (Westin et al., 1958; Callaghan et al., 1965; De Bie et al., 2021a). However, in recent years, several research teams have demonstrated the feasibility of supporting preterm goat and sheep fetuses with a variety of pumpless and pumped arteriovenous and venovenous AP systems using different approaches to establishing vascular access (Westin et al., 1958; Callaghan et al., 1965; Zapol et al., 1969; Reoma et al., 2009; Miura et al., 2012, 2016; Gray et al., 2013; Schoberer et al., 2014; Partridge et al., 2017; Usuda et al., 2017, 2019; Church et al., 2018a, 2018b; El-Sabbagh et al., 2018; Hornick et al., 2018; Coughlin et al., 2019). Three research teams have successfully demonstrated physiologic fetal sheep hemodynamics with normal organ maturation and minimal injury for periods of up to 1 month on pumpless arteriovenous AP systems (Partridge et al., 2017; Usuda et al., 2017, 2019; Church et al., 2018a, 2018b; Hornick et al., 2018).

Despite these advances, AP is yet to be proven in other animal models of the extremely preterm human infant, such as the pig. At a comparable stage of lung development to a previable human fetus, the preterm sheep is approximately twice the weight (Schittny, 2017; Morrison et al., 2018) whereas the pig is similar in size to the human (Eiby et al., 2013; Charest-Pekeski et al., 2021; Darby et al., 2021). Fetal size determines important anatomical considerations for establishing an extracorporeal membrane oxygenation (ECMO) system such as blood vessel diameter and blood pressure. In addition, fetal sheep possess two umbilical arteries (UA) and two umbilical veins (UV). This may allow a more stable transition to the AP circuit using one pair of UA and UV while maintaining native gas exchange *via* the placenta using the other pair of umbilical vessels, thus minimizing the cessation of oxygenation from the mother (Charest-Pekeski et al., 2021; Darby et al., 2021). By contrast, human and pig fetuses usually have one UV and two UAs, which may represent a more technically challenging approach to the initiation of AP support.

We have previously proposed (Charest-Pekeski et al., 2021) that Yucatan miniature pigs delivered at approximately 95 days gestation represent a realistic model of human infants born at the lower limit of viability in terms of their body weight and stage of lung development (Schittny, 2017). We recently demonstrated the feasibility of cannulating the umbilical vessels of preterm minipigs and transferring them to an AP system consisting of a warm, fluid environment and a pumpless ECMO circuit comprising a commercial neonatal oxygenator (Charest-Pekeski et al., 2021). However, our experiments were characterized by subphysiologic circuit flows, tachycardia, and the development of hydrops. Echocardiography further revealed evidence of diminished right ventricular function, which we attributed to excessive ventricular afterload resulting from the large priming volume of the circuit and small umbilical cannulas (Charest-Pekeski et al., 2021). Similar observations of heart failure occurring in pumpless arteriovenous AP systems have also been reported in sheep models of AP support (Reoma et al., 2009; Arens et al., 2011; Schoberer et al., 2014). The early development of AP technology typically incorporated pumps in the circuits, with the most successful experiments lasting several weeks, albeit with a requirement for continuous muscle relaxation to minimize oxygen consumption (Kuwabara et al., 1987; Unno et al., 1993; Unno et al., 1998). We hypothesized that the addition of a pump might improve the hemodynamics of our animals on the circuit by reducing afterload through the generation of negative pressure downstream of the umbilical arterial cannulas, thereby improving venous return and cardiac output. Herein, we sought to investigate the hemodynamics of a pumped AP system, comparing our findings to those obtained using the pumpless circuit (Charest-Pekeski et al., 2021).

## Results

### The Addition of a Centrifugal Pump Improves Successful Transition to AP and Support Times

**FIGURE 1:**
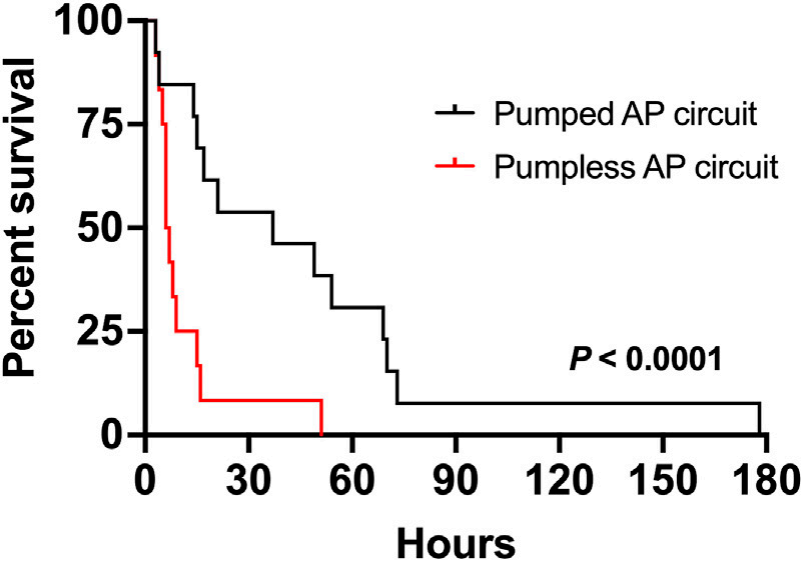
Comparison of duration of support of fetal pigs supported on a pumped and pumpless (Charest-Pekeski et al., 2021) AP circuit. Fetal pigs maintained using a pumped AP circuit are shown as a solid black line (*n* = 13), and fetuses supported using a pumpless circuit as the solid red line (*n* = 12).

Nineteen sows and 89 fetal pigs were used for pumped AP experiments (Table 1). A total of 32 fetal pigs were cannulated and transitioned to the AP. Types of perioperative complications preventing us from maintaining AP support were similar to those experienced in the pumpless AP circuit group and included the inability to establish circuit flows upon connection to the circuit (*n* = 11), accidental decannulation (*n* = 4), persistent vasospasm of the umbilical cord (*n* = 3), and excessive spiralling of the umbilical cord, which prevented cannulation or adequate cannula position (*n* = 1; Table 1). We successfully maintained 13 fetal pigs at an initial GA of 102 ± 4 days (range = 93–107 days) with a body weight of 616 ± 139 g (range = 390–820 g) on a pumped AP circuit for 46.4 ± 46.8 h (range = 3.4–177.8 h). This represented a significant improvement in the duration of support over the 12 fetal pigs (98 ± 4 days GA; 743 ± 350 g; 11 ± 13 h) maintained using a pumpless AP circuit (*p <* 0.0001; Table 1 and Figure 1). There was also a marked improvement in the rate of successful AP runs per litter using a pumped AP circuit compared to the pumpless AP circuit (62% pumped AP vs. 28% pumpless AP; Table 1). Successfully supported fetuses were similar in weight and GA in both pumped and pumpless groups (616 ± 139 g vs. 743 ± 350 g, *p* = 0.41; 102 ± 4 days vs. 98 ± 4 days, *p* = 0.24). On the pumped and pumpless AP circuits, fetal body weights were similar between successfully and unsuccessfully supported animals (616 ± 139 g vs. 523 ± 145 g, *p* = 0.47; 743 ± 350 g vs. 644 ± 184 g, *p* = 0.53). However, unsuccessfully supported fetuses or fetuses that we did not attempt to cannulate were significantly smaller in the pumped group compared to the pumpless group (523 ± 145 g vs. 644 ± 184 g, *p* = 0.04; 557 ± 124 g vs. 892 ± 362 g, *p* < 0.0001). Fetal weight at the time of termination of the AP study was significantly higher compared to pre-cannulation weight. Fetal weight gain correlated strongly with length of AP support (*r* = 0.89), with weight at the end of the experiment exceeding the expected growth trajectory of these fetuses. Individual data on supported fetuses, support times, and reasons for termination are shown in Table 2.

**TABLE 1:** Summary of fetal pigs from pumped and pumpless AP circuits (Charest-Pekeski et al., 2021).

	Pumped AP circuit				Pumpless AP circuit			
Parameter	Intervention				Intervention			
	Maintained on AP		Cannulated but not maintained on AP	Did not attempt to cannulate	Maintained on AP		Cannulated but not maintained on AP	Did not attempt to cannulate
N	13	AP success rate per litter (%)	19	57	12	AP success rate per litter (%)	56	9	p-values: pumpless vs. pumped AP circuit (ANOVA results)		
		62				28								
									Circuit type	Intervention	Interaction
Body weight (g)	616 ± 139 (390–890)		523 ± 145 (244–710)*	557 ± 124 (372–941; *n* = 24)*	743 ± 350 (500–1500; *n* = 7) ***ab***		644 ± 184 (343–1390; *n* = 52) ***a***	892 ± 362 (531–1470) ***b***	<0.0001	0.002	0.02
GA (days)	102 ± 4 (93–107)		103 ± 4 (93–108)	102 ± 5 (93–108)	98 ± 4 (93–107)		101 ± 7 (93–112)	101 ± 7 (97–110)	0.04	0.34	0.60

Two-way ANOVA. Multiple comparisons for fetal body weight and GA within pumped and pumpless AP circuits and between the intervention groups: ***ab*** *p* < 0.05. *p < 0.05 body weight is significantly different between pumped and pumpless AP circuits within intervention groups (i.e., body weight maintained on a pumped AP circuit vs. body weight maintained on a pumpless AP circuit). Interpretation of numbers for multiple comparisons: *a* represents a statistically significant difference compared with *b*.

**TABLE 2:** Summary data of 13 fetal pigs successfully cannulated and maintained using a pumped AP circuit. Data are presented by increasing GA and are expressed as mean ± SD. Δ Weight for fetal pigs was only calculated for animals that survived ≥24 h on the pumped AP circuit.

GA before (days)	GA after (days)	Duration on AP (hours)	Weight before (g)	Weight after (g)	Δ Weight (g)	Sex (M, F)	Reason for termination	Oxygenator
93	95	36.7	390	450	60	F	Fetal movement occluded UV flow	P
98	98	3.4	450	450	E	M	Heart failure	P
100	101	14.1	505	NA	NA	M	Mechanical failure (pump)	P
100	101	15.1	520	620	E	M	Heart failure	Q
100	102	49.4	590	750	160	F	Heart failure	P
101	104	70.0	762	1190	428	M	Mechanical failure (oxygenator)	Q
102	109	177.8	480	1176	696	M	Hydrops, cardiac dysfunction	P
105	108	68.5	610	NA	NA	NA	Mechanical failure (oxygenator)	P
105	105	17.1	676	NA	E	M	Fetal movement occluded UV flow	P
105	108	53.5	690	NA	NA	M	Fetal movement occluded UV flow	P
105	105	4.2	730	730	E	M	Thrombosis development in circuit	P
105	106	21.1	780	879	E	M	Equipment failure (sweep-gas supply)	P
107	110	72.5	820	876	56	F	Fetal movement occluded UV flow	P
**102 ± 4**	**104 ± 4**	**46.4 ± 46.8**	**616 ± 139**	**792 ± 272 (*n* = 9)**	**280 ± 277 (*n* = 5)**			

The bold letters represent MEAN ± SD.

NA, not available; M, male; F, female; GA, gestational age; E, excluded from Δ weight analysis; P, Paragon neonatal commercially available ECMO oxygenator; Q, Quadrox-I commercially available bypass oxygenator.

### Pumped AP, Circuits Achieve Higher Umbilical Venous Flow

**FIGURE 2:**
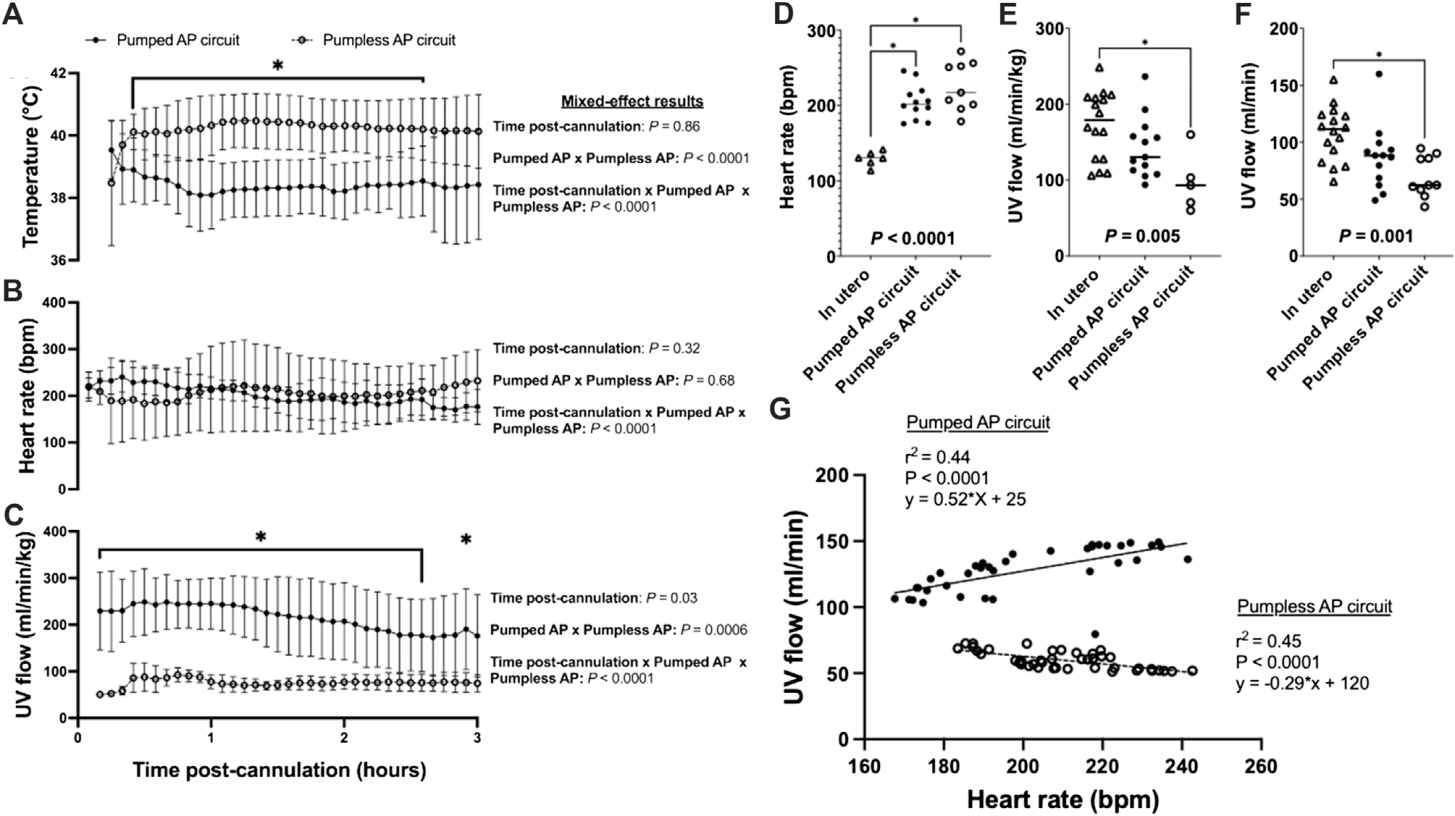
Fetal temperature, HR, and UV flow data for *in utero*, pumped and pumpless AP fetal pigs (Charest-Pekeski et al., 2021). **(A)** Temperature; **(B)** Fetal HR; and **(C)** UV flow vs. time post-cannulation. **(D)** Mean fetal HR; **(E)** mean indexed UV flow; and **(F)** mean absolute UV flow over the entire duration of support. **(G)** Correlations between HR and absolute UV flow for fetal pigs supported using a pumpless and pumped AP circuit. Fetal pigs maintained on a pumpless AP are represented as open circles and dashed lines (*n* = 10 for temperature; *n* = 9 for HR; *n* = 5 for UV flow), and fetal pigs maintained on a pumped AP circuit (*n* = 13 for temperature and UV flow; *n* = 12 for HR) as black circles and solid line. *In utero* fetal pigs are represented as open triangles (*n* = 6 for HR; *n* = 16 for indexed and absolute UV), pumpless AP fetal pigs as open circles (*n* = 10 for absolute UV flow) and pumped AP fetal pigs as black circles (*n* = 13 for indexed UV flow).

**FIGURE 3:**
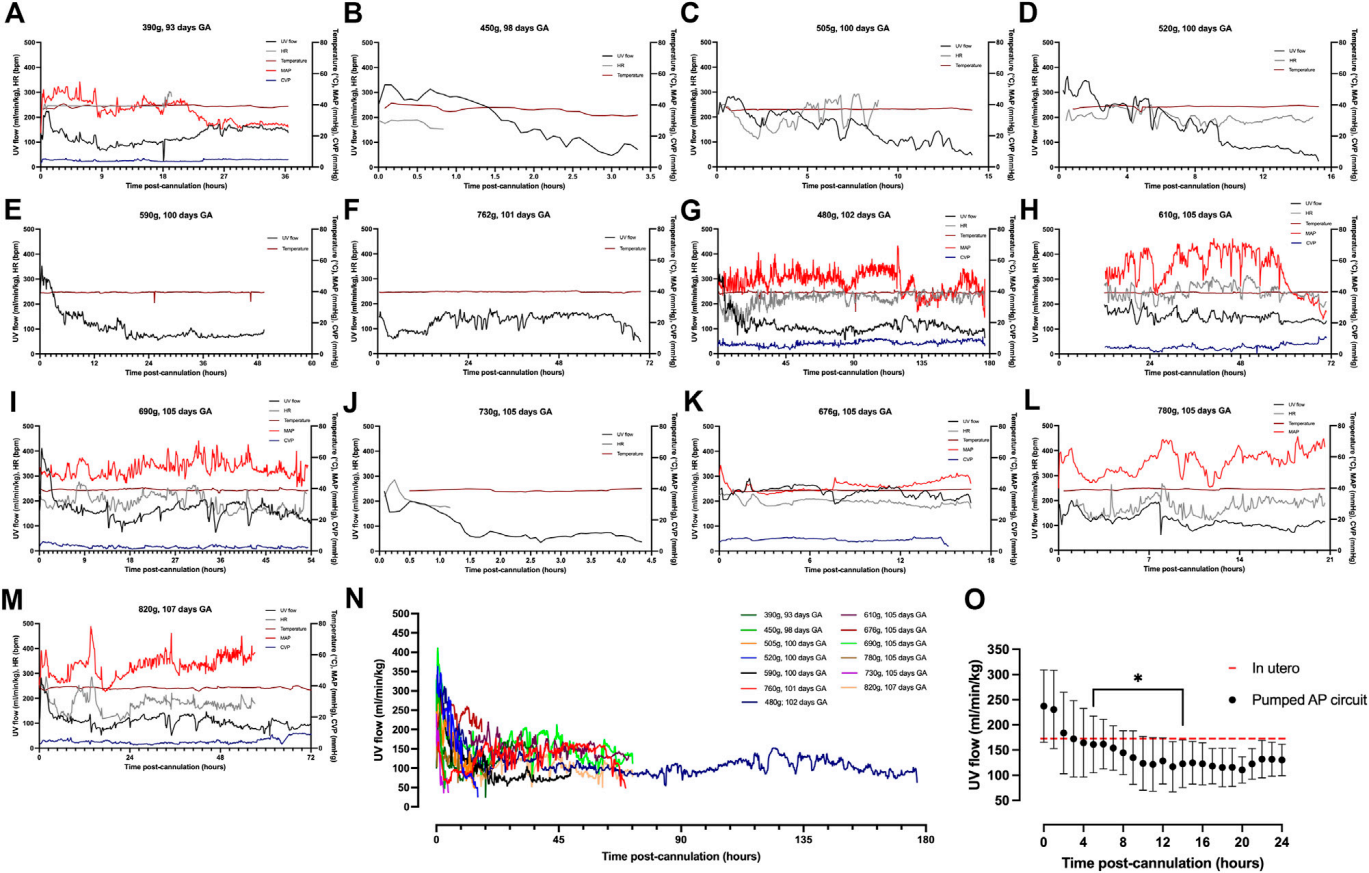
Hemodynamic data in pumped AP fetal pigs. Data from *n* = 13 fetal pigs. **(A–M)** Changes in UV flow, HR, temperature, MAP, and CVP over time for each individual animal supported using a pumped AP circuit. **(N)** Individual UV flow patterns for fetal pigs. **(O)** Aggregate UV flow data for pumped AP fetal pigs. Pumped AP fetal pigs are represented as black circles, and in utero UV flow as the dashed red line. Data are presented in 5-minute **(A–N)** and 1-hour epochs **(O)**. * Significantly different from the first recorded data point.

**FIGURE 4:**
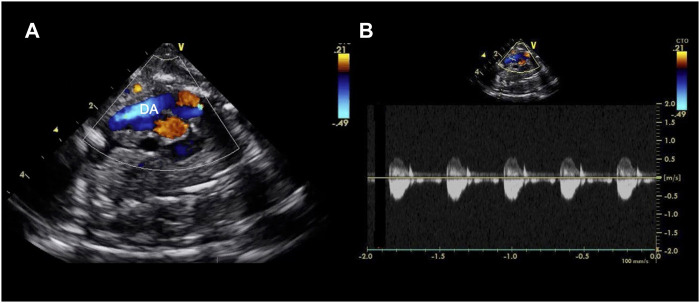
Echocardiographic axial mediastinal image of a fetal pig maintained using a pumped AP circuit, demonstrating the ductus arteriosus (DA) widely patent **(A)** and with a normal low velocity flow profile as shown on the pulsed wave doppler **(B)**. A video can be found in the Supplementary Material.

Animals on a pumped AP circuit maintained a physiologic core body temperature throughout the experiment. The temperature was lower in fetal pigs supported on a pumped system than pigs supported on a pumpless system (*p* < 0.0001, Figure 2A). UV flow rates of fetuses supported by the pumped AP circuit were comparable to *in utero* controls, while UV flow on the pumpless AP system was subphysiologic (*in utero*; 108 ± 24 ml/min vs. pumped; 87 ± 28 ml/min vs. pumpless; 70 ± 18 ml/min, *p* = 0.001; *in utero*; 173 ± 45 ml/min/kg vs. pumped; 143 ± 40 ml/min/kg vs. pumpless; 97 ± 39 ml/min/kg, *p* = 0.005; Figures 2C,E,F). Absolute UV flow measured within the first 3 h post-cannulation was positively correlated to HR in pumped AP circuits (*r* ^*2*^ = 0.44; *p* < 0.0001; Figure 2G), but negatively correlated in fetuses maintained on a pumpless circuit (*r* ^*2*^ = 0.45; *p* < 0.0001; Figure 2G), with differences between the slopes being extremely significant (*p* < 0.0001). Figures 4A–M demonstrate the changes in UV flow, HR, temperature, MAP, and CVP over time for the 13 fetal pigs successfully maintained using a pumped AP circuit. Upon initiation of AP support, we observed supraphysiologic UV flow, despite a low pump rate setting (Figures 3A–N). In longer trial runs, this period of supraphysiologic UV flow persisted for approximately 6 h, then decreasing to subphysiologic flow, plateauing at ∼12 h of support (Figure 3O). In several experiments (*n* = 8/13) excessive spasmodic fetal movements resulted in a complete cessation of UV flow and consequently led to the termination of the experiment (Table 2). In four studies, development of a thrombosis in one of the UAs in the first 12 h of support was associated with a significant reduction in UV flow. However, clot formation in the AP circuit was infrequent with mean activated clotting times of 247 ± 62 s (*n* = 12) and only one fetus terminated due to development of thrombosis (Table 2).

**FIGURE 5:**
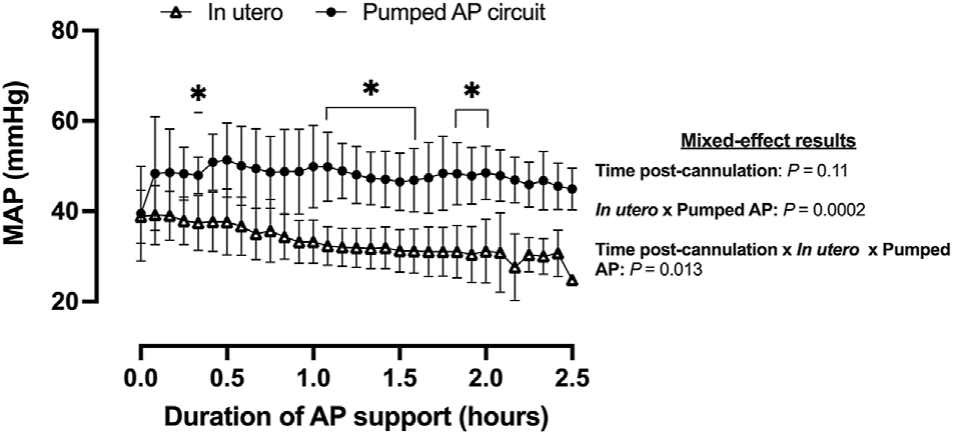
MAP in fetal pigs studied *in utero* and maintained using a pumped AP circuit. Fetal pigs studied *in utero* are represented in open triangles (*n* = 21) and fetal pigs maintained using a pumped AP as black circles (*n* = 6). Data are presented as 5-min epochs.

**FIGURE 6:**
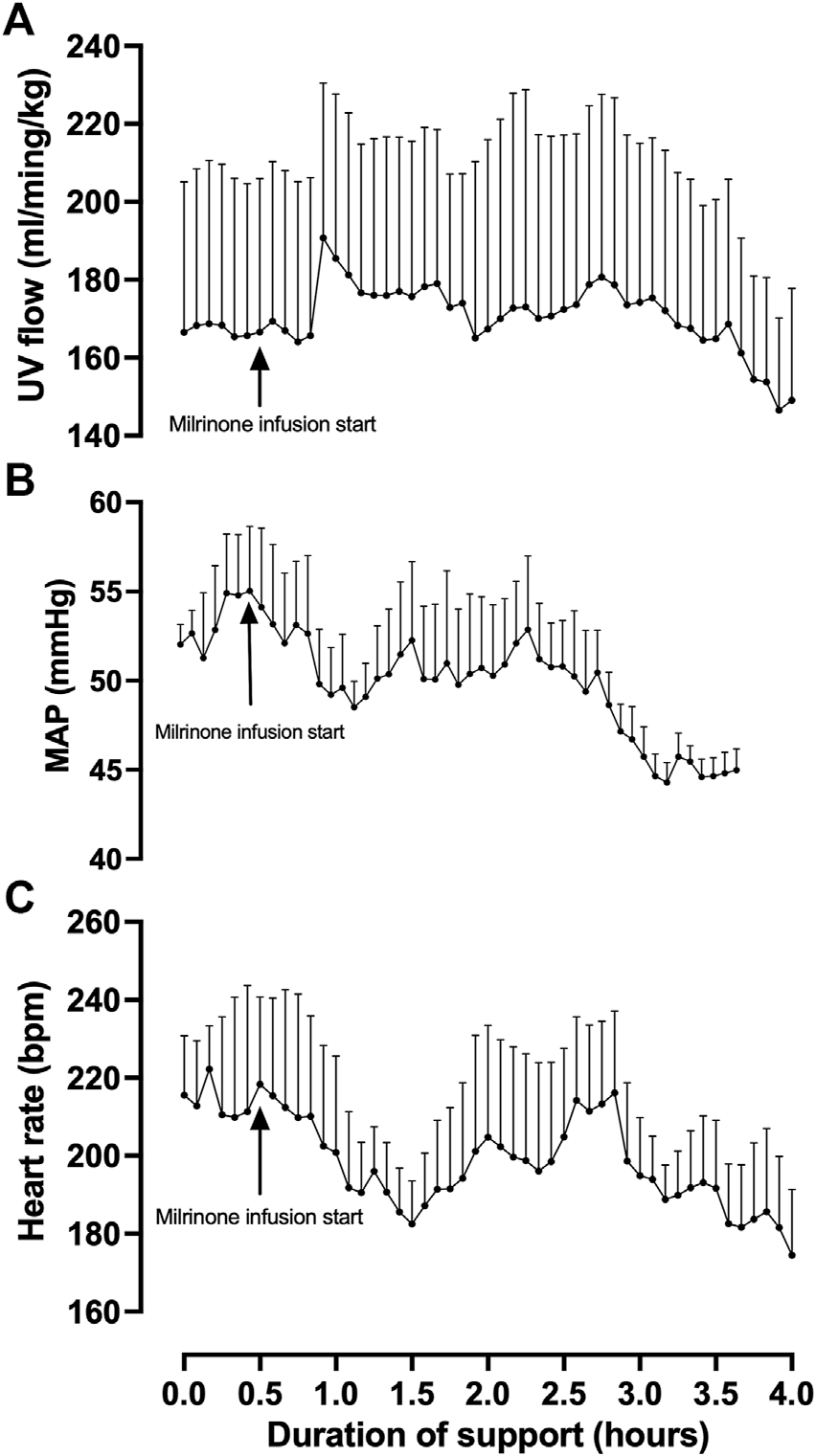
Effects of milrinone lactate infusion on fetal pig indexed UV flow **(A)**, MAP **(B)**, and HR while maintained using a pumped AP circuit. Maintenance infusion of milrinone lactate was used in successful AP experiments (*n* = 5) for hemodynamic support. “Milrinone infusion start” indicates the time point at which milrinone lactate IV infusion into the circuit began. Data are presented as 5-minute epochs and expressed as mean ± SEM.

Fetal pigs were tachycardic for a large proportion of the AP studies (Figures 2B,D; and Figures 4A–M). Both pumped (205 ± 28 bpm; *p <* 0.0001; Figure 2D) and pumpless (206 ± 38 bpm; *p <* 0.0001; Figure 2D) fetal pig groups were similarly tachycardic throughout the experiment compared to *in utero* controls (130 ± 10 bpm; Figure 2D). Compared to *in utero* control animals, fetal pigs were hypertensive on the pumped AP system (*p* = 0.013; Figure 5). Approximately 20 min after initiation of a milrinone (a phosphodiesterase-3 inhibitor) infusion, there was a marked increase in UV flow, followed by a steady decline over a 3 h period (Figure 6A). MAP and HR decreased following the start of the milrinone infusion, which persisted for ∼1 h before returning to a steady state (Figures 6B,C).

**FIGURE 7:**
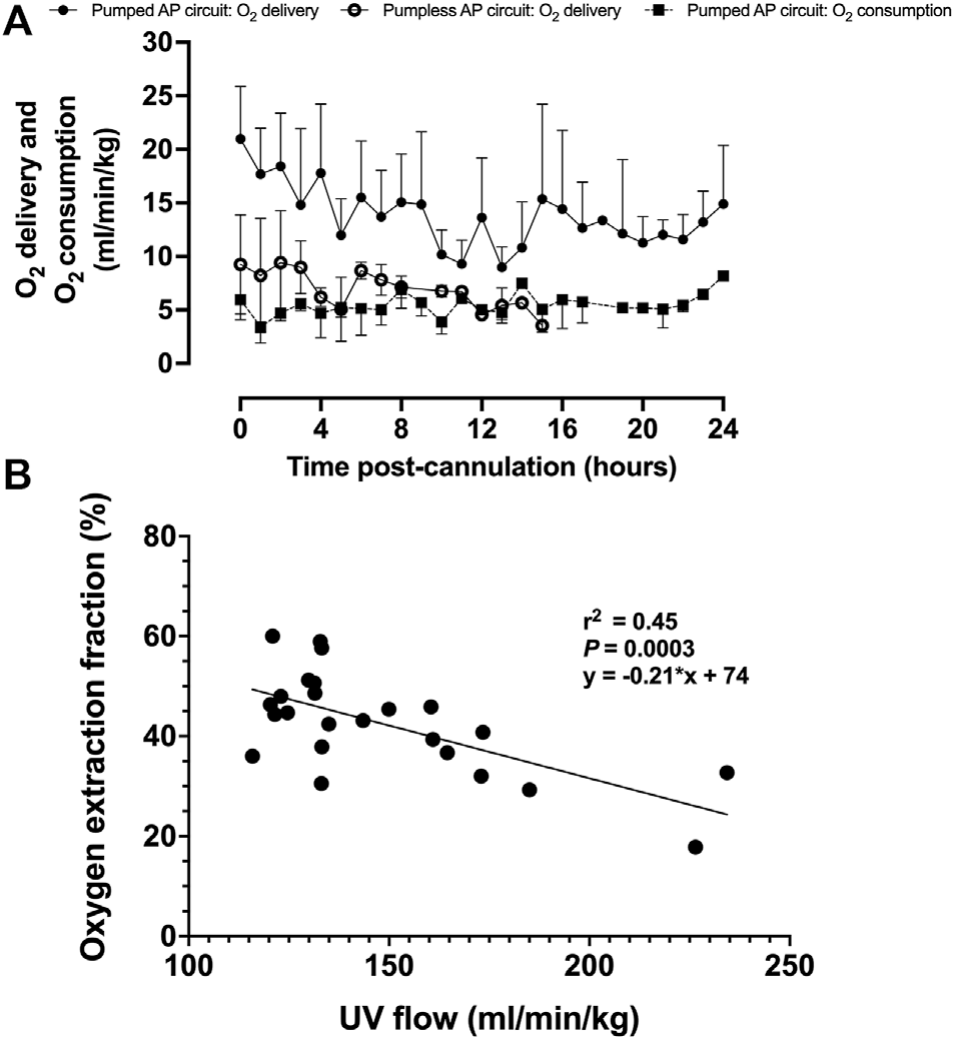
Fetal oxygen delivery, oxygen consumption, and oxygen extraction fraction on a pumped AP circuit. **(A)** Fetal oxygen delivery on the pumped AP circuit is represented as black circles (*n* = 13) and open circles for the pumpless AP circuit (*n* = 5) (Charest-Pekeski et al., 2021), whereas oxygen consumption is shown as black squares with a dashed line (*n* = 11). **(B)** Correlation between UV flow and oxygen extraction fraction (*n* = 11). Data are presented as 1-h averages over the first 24 h of AP support.

Upon initiation of AP support, fetal oxygen delivery was physiologic (Rudolph, 2009; Acharya et al., 2016). We observed a decline in oxygen delivery and increase in oxygen consumption over time (Figure 7A). UV flow and oxygen extraction fraction correlated negatively (*r* ^*2*^ = 0.45; *p* = 0.0003; Figure 7B).

### Fetal Blood Gas Analysis Indicates Supraphysiologic Oxygen Tension and Fetal Anemia

**FIGURE 8:**
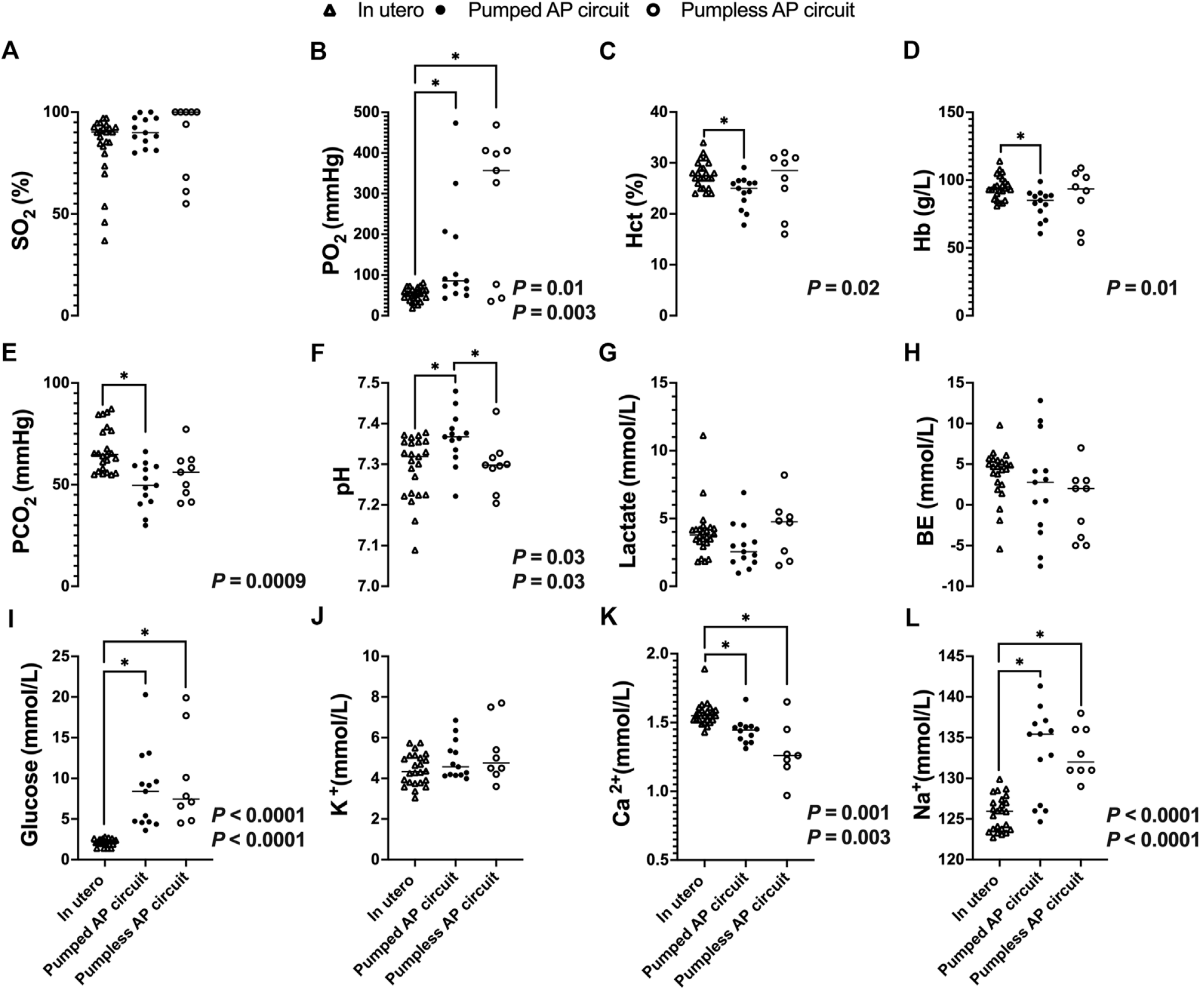
Blood gases, electrolytes, lactate, and glucose concentrations in fetal pigs studied in utero versus fetal pigs supported using a pumped and pumpless AP circuit (Charest-Pekeski et al., 2021). In utero fetal pig data represented by open triangles (*n* = 24), fetal pigs supported on a pumped AP circuit as black circles (*n* = 13), and fetuses supported on a pumpless AP circuit as open circles (*n* = 9). **(A)** SO_2_, **(B)** PO_2_, **(C)** Hct, **(D)** Hb, **(E)** PCO_2_, **(F)** pH, **(G)** Lactate, **(H)** BE, **(I)** Glucose, **(J)** K^+^, **(K)** Ca^2+^, and **(L)** Na+. If two *p-*values are presented, the first *p-*values represents the pumped AP circuit, and the second *p-*value represents the pumpless AP circuit. Data for pumped and pumpless AP circuits are presented as an average for the entire duration of support.

While there was no significant difference in SO_2_ between fetal pigs studied *in utero* and those maintained using a pumped or pumpless AP circuit (Figure 8A), both AP circuits resulted in a significantly higher PO_2_ compared to *in utero* controls (*in utero*; 53 ± 16 mmHg vs. pumped; 141 ± 129 mmHg, *p* = 0.0001, vs. pumpless; 280 ± 176 mmHg, *p* = 0.003, Figure 8B). Conversely, PCO_2_ was significantly lower in the pumped AP group compared to *in utero* controls (*in utero*; 67 ± 11 mmHg vs. pumped; 49 ± 11 mmHg, *p* = 0.0009, Figure 8E). Animals on the pumped AP were significantly more alkalotic than both pumpless AP fetuses and *in utero* controls (*in utero*; 7.29 ± 0.08 vs. pumped; 7.36 ± 0.07, *p =* 0.03, vs. pumpless; 7.30 ± 0.06, *p* = 0.03, Figure 8F). BE and lactate were similar between groups (Figures 8G,H). The pumped AP group had lower hematocrit (*in utero*; 28 ± 3%; vs. pumped; 24 ± 3%, *p* = 0.02, Figure 8C) and hemoglobin (*in utero*; 96 ± 9 g/L vs. pumped: 82 ± 11 g/L, *p* = 0.01, Figure 8D), as well as higher Na^+^ in both AP supported groups compared to *in utero* fetuses (*in utero*; 126 ± 2 mmol/L vs. pumped; 133 ± 5 mmol/L, *p* < 0.0001; vs. pumpless; 133 vs. 3 mmol/L, *p* < 0.0001, Figure 8L). Ca^2+^ concentrations were subphysiologic in both pumped and pumpless groups (*in utero*; 1.56 ± 0.087 vs. pumped; 1.44 ± 0.092 mmol/L, *p =* 0. 003, vs. pumpless; 1.29 ± 0.21 mmol/L, *p =* 0. 001, Figure 8K). Blood glucose, which was administered continuously either directly or as part of total parenteral nutrition in both AP groups, was significantly higher in both AP groups, compared to *in utero* controls (*in utero*; 2.1 ± 0.43 mmol/L vs. pumped; 8.5 ± 4.8 mmol/L, *p* < 0.0001, vs. pumpless; 9.8 ± 5.8 mmol/L, *p* < 0.0001, Figure 8I). There was no difference in K^+^ concentrations between *in utero* fetal pigs and those maintained using a pumped and pumpless AP circuit (Figure 8J).

## Discussion

To our knowledge, this is the first study to examine the hemodynamics and blood gas status of preterm pigs supported using an AP system incorporating a small centrifugal pump. We previously showed the feasibility of supporting our animal model using a pumpless AP circuit connected to the fetal circulation *via* the umbilical arteries and vein (Charest-Pekeski et al., 2021); however, our experiments were characterized by significant hemodynamic decompensation within hours. We attributed this to afterload imbalance, elicited by supraphysiologic circuit resistance and further exacerbated by impaired umbilical venous return, which we surmised resulted in higher sympathetic tone, further exacerbating the increase in afterload (Charest-Pekeski et al., 2021). Although other research groups have highlighted potential limitations of pumped ECMO circuits, including an increased risk of pump-induced hemolysis (Partridge et al., 2017), afterload imbalance, myocardial strain, and impaired autoregulation of UV blood flow (Kuwabara et al., 1987), we hypothesized that the addition of a small pump may reduce right ventricular afterload and sustain adequate UV flow, thereby improving survival on the AP. Prior studies using a pumped veno-venous AP system in preterm sheep have demonstrated stable hemodynamics and normal brain and pulmonary development (Church et al., 2018b; 2018a). The present study examined differences in hemodynamics, blood gases, electrolytes, biochemistry, and survival of fetal pigs supported on a pumped versus pumpless AP system (Charest-Pekeski et al., 2021). The increase in AP support to 46.4 ± 46.8 h represented a significant increase in survival over fetal pigs supported without a pump, possibly due to more reliable and physiologic UV flow compared to a pumpless system. Despite the pump support, following a period of supraphysiologic UV flow, the animals continued to experience a slower but consistent decline in UV flow and the development of signs of circulatory deterioration.

Partridge et al. demonstrated AP support of fetal sheep weighing 1–2 kg using a pumpless system for up to 28 days without the use of vasopressors (Partridge et al., 2017). Using a similar AP circuit, Usada et al. reported survival of fetal sheep for up to 1 week (Usuda et al., 2017). To investigate the potential utility of AP systems for extremely preterm infants born at 22–25 weeks GA, two research groups cannulated sheep born at 85–95 days GA weighing ∼0.5–0.8 kg. Although cannulation was technically feasible, one study reported the development of hydrops fetalis following 5–8 days of support (Hornick et al., 2019), whereas the other required the use of aggressive pharmacological interventions to achieve hemodynamic stability (Usuda et al., 2019). Although fetal sheep delivered at 85–95 days GA share similar body weight to human fetuses delivered at the biological limit of viability, they are developmentally immature and their pulmonary development is analogous to a human fetus delivered at 18 weeks GA (Eiby et al., 2013; Morrison et al., 2018; Chan et al., 2020; De Bie et al., 2021a). By contrast, fetal minipigs delivered at 93–107 days GA weighing 0.39–0.82 kg are in the canalicular and saccular stages of lung development and comparable to preterm human fetuses delivered at 22–28 weeks GA. Thus, fetal pigs delivered at 95 days GA would therefore be equivalent in body weight and lung maturity to human fetuses born at 22–25 weeks GA, and may represent a more appropriate model for the development of an AP system (Eiby et al., 2013; Chan et al., 2020; Charest-Pekeski et al., 2021).

Upon initiation of AP support, we observed supraphysiologic UV flow in all pumped AP experiments. Umbilical vein blood flow was higher in fetuses supported with a pumped circuit than in fetuses supported using a pumpless system. Our experiments revealed a positive correlation between fetal HR and UV flow on the pumped AP, with persistent tachycardia seen with both systems. Contrary to our speculations regarding supraphysiologic resistance in the pumpless circuit (Charest-Pekeski et al., 2021), we propose that the negative pressure generated by the centrifugal pump may have diminished right ventricular afterload and minimized resistance to flow across the UA cannulas, resulting in higher than normal flow rates. The association we observed between fetal HR and UV flow in animals supported on the pumped circuit may be attributable to the Frank-Starling mechanism and Anrep effect (von Anrep, 1912), whereby increased ventricular preload enhances myocardial stretch and tension, resulting in increased contractility. Although this relationship holds true for slight increases in end-diastolic filling pressures, the fetal heart has little preload reserve (Kirkpatrick et al., 1976; Gilbert, 1980; Thornburg and Morton, 1986; Rychik, 2004). Thus, further augmentation of cardiac output and contractility are also driven by the Bowditch effect (Piot et al., 1996) through increases in fetal HR (Kiserud and Acharya, 2004). Therefore, we speculate that these supraphysiologic circuit flows lead to increased preload, tachycardia, sympathetic nervous system activation, and likely higher cardiac output within the first hours of AP support.

**FIGURE 9:**
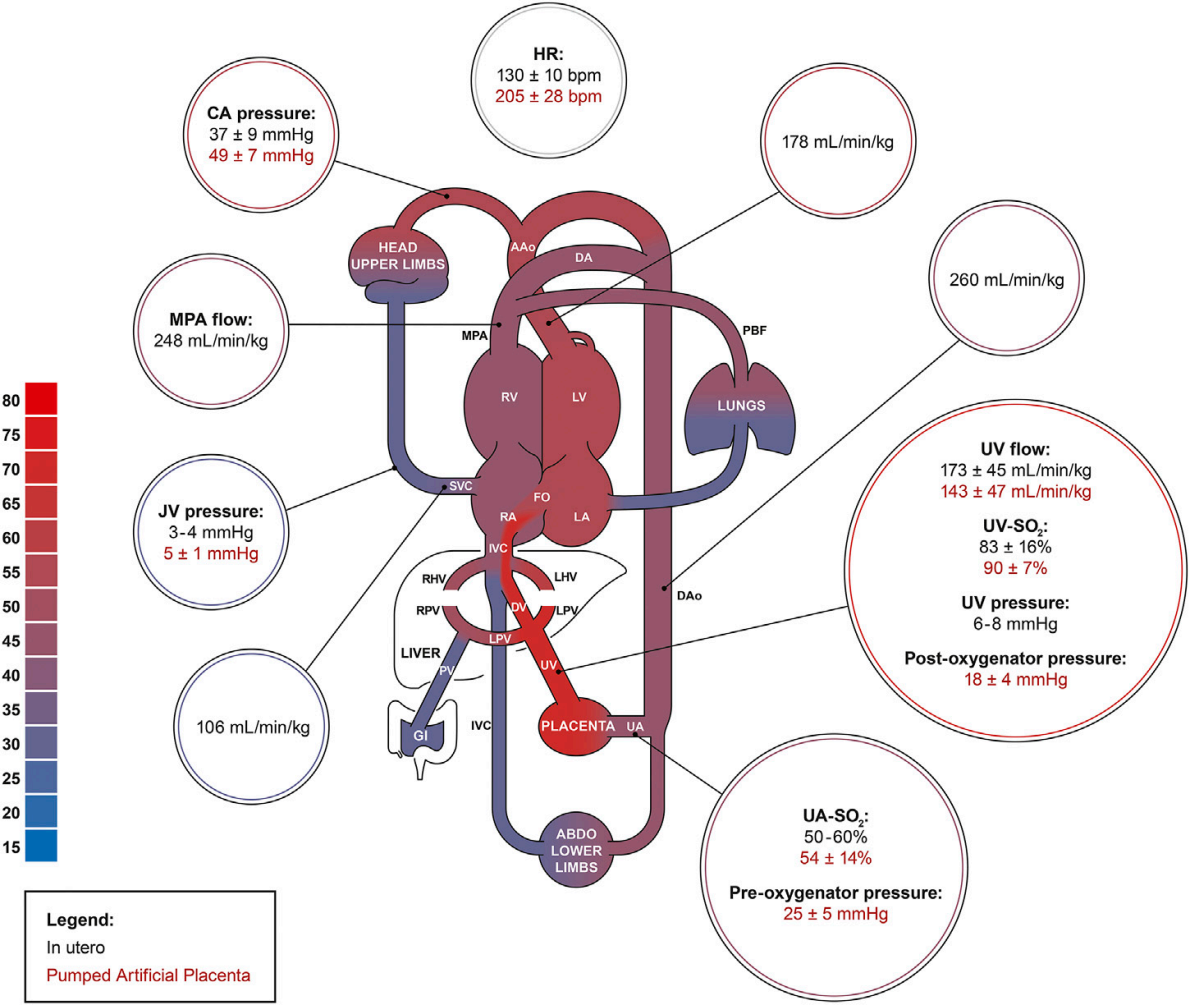
The fetal pig circulation. Comparing physiologic in utero values (black; *n* = 61) to data acquired from the pumped AP experiments (red). Physiologic in utero data was acquired from Rudolph et al., Acharya et al., Charest-Pekeski et al., and Berman et al. (Rudolph and Heymann, 1970; Berman et al., 1976; Rudolph, 2009; Acharya et al., 2016; Charest-Pekeski et al., 2021).

After ∼24 h of AP support, we observed marked reductions in UV flow compared to the high flows seen at the start of AP support and in many experiments, UV flows then remained subphysiologic. The drop in UV blood flow and persistent tachycardia could be explained by increased sympathetic nervous activation and continuous peripheral vasoconstriction. Although the introduction of milrinone improved UV flow and reduced BP, its inotropic and systemic vasodilatory effects appeared to be short-lived, indicating the presence of overwhelming perturbation of fetal cardiac loading conditions. However, our findings suggest that phosphodiesterase inhibitors may have a positive impact on the circulation of fetal animals supported on the AP (Usuda et al., 2019; De Bie et al., 2021b). Despite similar SO_2_, blood gases showed higher PO_2_, lower PCO_2_, as well as a more alkalotic pH in fetuses on the pumped AP compared to *in utero* controls. We were able to manage O_2_ uptake and CO_2_ elimination better on the pumped circuit than pumpless. However, the gas exchange capacity of the oxygenator used in both groups easily outperforms the native placenta, resulting in supraphysiologic UV PO_2_, and subphysiologic PCO_2_. High oxygen tension is a known contributor to umbilical cord and ductal constriction and may have negatively affected our experiments (Eltherington et al., 1968; McGrath et al., 1986). Furthermore, fetal hyperoxygenation is associated with pulmonary arterial vasodilation and dysregulation of fetal circulatory distribution which could supress normal fetal lung growth and maturation on the AP. High PO_2_ can also increase the generation of reactive oxygen species and increase the risk of oxidative stress to vulnerable fetal organ systems (Torres-Cuevas et al., 2017; Vali and Lakshminrusimha, 2017; Jiang et al., 2021). Excessive CO_2_ elimination and incorrect mixture of CO_2_ in the sweep gas may explain the respiratory alkalosis we observed in the pumped AP animals. Glucose was higher in the AP groups than *in utero* because we supplemented the fetuses with glucose. Persistent fetal hyperglycemia is associated with circulatory and electrolyte effects, tissue injury, cardiac hypertrophy, and macrosomia, and should be avoided in future experiments (Kc et al., 2015; Bogo et al., 2021). Nevertheless, it seems likely that the fetal deterioration we observed on both pumped and pumpless AP circuits was more likely to be a consequence of abnormal cardiac loading than fetal hyperglycemia. Although we observed no change in lactate between controls and animals on the AP, it should be noted that both glucose and lactate are higher in pigs than sheep fetuses (Darby et al., 2019, 2021; Aujla et al., 2021; Charest-Pekeski et al., 2021; Schrauben et al., 2021). Mean blood pressure in the UV is 6–8 mmHg in near-term sheep and human fetuses (Rudolph, 2009; Acharya et al., 2016). In our pumped AP studies, we observed pre- and post-oxygenator pressures of 25 mmHg and 18 mmHg, respectively (Figure 9). Although we did not measure blood pressure in the UV, it is likely that the pumped circuit exposed the UV to elevated blood pressure, even if there was some degree of pressure difference across the venous cannula. Berman et al. (1976) advanced balloon catheters into the common UV of fetal sheep and demonstrated marked reductions in placental blood flow with increasing UV pressures. This was attributed to a decreased pressure gradient across the umbilical circulation, resulting in diminished umbilical flows. We hypothesize that a similar phenomenon may be occurring in the early phase of our AP experiments, with UV constriction induced by the high pressures and shear stress resulting from the elevated post-pump pressures.

We observed a steady widening of the veno-arterial saturation gradient across the umbilical circulation during our AP runs that was negatively correlated with UV flow, indicating higher oxygen extraction at the tissues in the setting of a net reduction in UV flow and oxygen delivery. One factor that might additionally contribute to the progressive reduction in oxygen delivery was the low hemoglobin concentration we observed in animals supported on the pumped system. The large priming volume of the circuit and oxygenator contributes to fetal anemia because the circuit is primed with maternal blood. During pregnancy, maternal blood volume increases by ∼40% and there is a progressive decline in hemoglobin concentrations (Rockwell et al., 2003; Gonzales et al., 2009; Means, 2020). Conversely, fetal hemoglobin increases with GA to sustain fetal tissue oxygenation in the setting of declining PO_2_ (Jopling et al., 2009; Mari et al., 2015; Acharya et al., 2016). Thus, the mixing of maternal and fetal blood in the AP circuit contributes to hemodilution of fetal blood, while ongoing blood sampling further depletes fetal red blood cells. At the low oxygen tensions present in fetal tissues, maternal hemoglobin, which has lower affinity for oxygen than fetal hemoglobin, would be expected to result in greater oxygen delivery to the tissues. Under normal physiologic conditions, the greater affinity of fetal hemoglobin for oxygen is important because it promotes oxygen transport from the maternal to fetal circulation at the placenta. However, we would suggest that this mechanism is likely to be less significant in the setting of AP support, where oxygen transport occurs very readily from the gas in the oxygenator across the membrane into fetal blood and so the oxygenation of the umbilical circulation is therefore less dependent on fetal hemoglobin (Charest-Pekeski et al., 2021). Thus, the lower oxygen affinity of maternal hemoglobin may help to offset any adverse effect of the anemia caused by our use of maternal blood, which has a lower hematocrit than fetal blood, for priming our circuit and providing top up transfusions. This conclusion is supported by our blood gas data, which are not suggestive of the metabolic acidosis that would be expected in the setting of inadequate tissue oxygen delivery. To circumvent fetal anemia in future experiments, it may be possible to hemoconcentrate the circuit prime. Furthermore, pump-induced hemolysis may have also contributed to fetal anemia in these experiments. Other groups have recommended the administration of daily doses of erythropoietin to minimize the need for successive maternal donor blood transfusions (Partridge et al., 2017). Excessive CO_2_ elimination and incorrect mixture of CO_2_ in the sweep gas may explain the respiratory alkalosis we observed in the pumped AP animals. Miniaturization of the oxygenator and pump head could also help to reduce fetal anemia by reducing circuit volume.

### Study Limitations

There are several limitations of the present study. Firstly, we have not yet characterized the etiology of the variation in UV flows on the pumped AP circuit. We speculate that a combination of UV constriction and supraphysiologic UV pressures, as well as supraphysiologic oxygen tension may be contributing to progressively diminished UV flows. However, this hypothesis should be tested with direct measurements of UV pressure. Similarly, future experiments would be strengthened by the simultaneous measurement of umbilical artery pressure. We speculate that adrenergic drive may be contributing to tachycardia and peripheral vasoconstriction on the AP that would be enhanced by the measurement of circulating catecholamines. Secondly, although the addition of a centrifugal pump to the AP improved fetal pig hemodynamics, we periodically encountered air-entrainment into the circuit, as well as cavitation of fetal blood at excessively high negative pressures, resulting in diminished circuit flows. Although we did not detect pump-induced hemolysis, this could be a limiting factor during long-term AP experiments and could explain the fetal anemia on the circuit. Fourthly, systemic inflammation may have also contributed to the hemodynamic instability and cardiovascular decompensation we observed in pumped AP fetal pigs as centrifugal pumps have been reported to induce low-level systemic inflammation (Ugaki et al., 2007). The analysis of inflammatory cytokines may be helpful to delineate the role of inflammation in fetal pig circulatory physiology on the AP. Additionally, we did not assess the brain, heart, and lungs for evidence of ischemic, embolic, thrombotic, and hemorrhagic events on the AP circuit: important steps in assessing organ health on the pumped and pumpless AP circuit. Finally, we compared *in utero* hemodynamic and blood gasses and biochemistry data from White Landrace cross piglets to the fetal Yucatan fetal pigs supported on the AP circuits. This could have impacted our study findings, as White Landrace cross piglets tend to be significantly larger than fetal Yucatan pigs.

### Future Directions

Recent developments in AP technology have enabled sustained extrauterine fetal life on a pumpless arteriovenous ECMO circuit. Of note, these experiments were conducted using customized hollow-fiber membrane oxygenators that are unavailable for commercial use. Our initial approach in developing a pumpless AP system involved cannulas with the largest luminal diameter to wall thickness ratio possible to minimize circuit resistance. Our failure to demonstrate hemodynamic stability using a pumpless circuit encouraged our team to explore the use of mechanical support in the extremely preterm fetal pig. While the addition of a pump to the circuit improved survival, we observed supraphysiologic circuit flows, evidence of adrenergic drive, and high cardiac output at the start of support, followed by diminished UV flows with persistent tachycardia and hypertension in keeping with diminished cardiac output. This pattern may indicate a progressive increase in circuit resistance resulting from constriction in the cord vessels. An alternative approach to normalizing UV pressures and minimizing the pressure difference across the UV cannula would be to introduce a pressure drop in the circuit through restriction of the circuit or cannula lumen. According to Hagan-Poiseuille’s law, reducing the calibre of the venous side of the circuit would increase circuit resistance and limit the supraphysiologic circuit blood flow seen at the onset of our AP experiments.

Miniaturization of the oxygenator and pump head may also help to achieve hemodynamic stability in our animal model. In sheep, placental blood volume at ∼116 days GA (term = 145 days) is ∼60–80 ml/kg, which represents ∼30–40 ml for a 0.5 kg fetus (Yao et al., 1969; Brace, 1983). The AP circuit used in the present study has a priming volume that is nearly double the expected placental blood volume for a fetal sheep delivered at 116 days gestation, which likely accounts for the increased afterload induced in the pumpless AP experiments, as well as contributing to fetal anemia. Reductions in the oxygenator membrane surface area may also reduce the need for excessive heparin administration. Thus, modifications to the circuit through miniaturization of the oxygenator and centrifugal pump may be helpful for establishing an AP system capable of supporting the long-term physiological requirements of extremely preterm fetal pigs. Future studies will include more detailed analysis of physiological parameters while on the circuit (e.g., echocardiography and blood pressure in different vessels), as well as measures of organ development (e.g., lung maturity and brain health).

## Conclusion

Over the past decade, important advances have been achieved in the field of artificial womb/AP technology. In experiments using a miniature pig model of the AP in which commercial neonatal ECMO oxygenators were connected to the fetal circulation *via* the umbilical cord we demonstrated a marked improvement in survival, UV flow, and oxygen delivery in circuits that incorporated a small centrifugal pump compared with animals maintained on a pumpless circuit. However, despite the addition of the pump, we observed a progressive fall in UV flow with persistent tachycardia and hypertension, which we attribute to preload imbalance, increased sympathetic tone, and UV hypertension. Thus, despite observing a clear short-term benefit with the addition of a centrifugal pump in supporting the fetal pig hemodynamics, we conclude that further modifications to the AP circuit are needed. A reduction to the size of the UV cannula could represent an alternative approach for mediating physiologic circuit flows and venous pressures. Given the limitations of neonatal intensive care therapies in preventing iatrogenic organ injury and neonatal death, we remain hopeful that the AP could provide reductions in the mortality and morbidity associated with preterm birth by maintaining a fetal circulation while allowing for normal growth and development of fetal organ systems.

## Materials and Methods

### Experimental Groups

The results presented in this report are comprised from fetal pigs studied 1) *in utero* and 2) while maintained using a pumped AP circuit and 3) of our previously published findings using a pumpless AP system (Charest-Pekeski et al., 2021). Fetal Large White Landrace pigs were studied *in utero* at 105 GA ± 5 (*n* = 45) and Yucatan miniature pigs at 107 ± 3 days (*n* = 16), providing reference physiologic data regarding heart rate (HR), blood pressure, blood gases, electrolytes, lactate and glucose concentrations, and UV flow (Charest-Pekeski et al., 2021). Fetal Yucatan miniature pigs (*n* = 13) were maintained using an umbilical arteriovenous AP circuit consisting of a centrifugal pump and oxygenator and using a pumpless AP circuit (*n* = 12) (Charest-Pekeski et al., 2021). We included AP subjects that survived on the system for a minimum of 3 h.

### Animals and Approvals

*In utero* BP, HR, blood gases, electrolytes, glucose, and lactate measurements were obtained in White Landrace Cross sows (*n* = 6; term = 115 days) at the Preclinical Imaging and Research Laboratories, South Australian Health and Medical Research Institute (SAHMRI). All procedures were approved by the SAHMRI Animal Ethics Committee (Charest-Pekeski et al., 2021). Sows were individually housed with environmental and social enrichment. The AP experiments were conducted in the Lab Animal Services (LAS) facility at The Hospital for Sick Children (SickKids), Peter Gilgan Center for Research and Learning in Toronto, Ontario. All maternal and fetal surgeries were approved by the SickKids Animal Care Committee and all procedures complied with the Canadian Council on Animal Care, Ontario Ministry of Agriculture, Food and Rural Affairs, Animals for Research Act guidelines, and the Care and Use of Animals for Scientific Purposes. Pregnant Yucatan miniature pigs (*n* = 46; term = 115 days) were acquired from Memorial University of Newfoundland, and Sinclair Bioresources and transported as per the Health of Animals Act of Canada (Charest-Pekeski et al., 2021). Yucatan pigs were housed in pairs for at least 2 weeks prior to surgery to increase socialization between the animals and allow for acclimation to human handling and to their new environment. Sows were provided with *ad libitum* food and water, and environmental enrichments as per SickKids standard operating procedures.

### Protocol for in Utero Studies

Large White Landrace Cross gilts (*n* = 6; 98 ± 7 days GA; term = 115 days) were anaesthetised with an intramuscular injection (I.M.) of 20 mg/kg ketamine and inhalation of isoflurane. Gilts were intubated and general anaesthesia was maintained using isoflurane with 2 L/min O_2_ and 4 L/min medical air. Gilts were positioned on the operating table on their backs, an incision was made along the abdomen, the uterus was incised, and a fetal head was exposed. Fetal pigs (*n* = 24) were cannulated *via* the UV, and venous blood was sampled for partial pressure of oxygen (PO_2_), partial pressure of carbon dioxide (PCO_2_), oxygen saturation (SO_2_), pH, hemoglobin (Hb), bicarbonate (HCO_3_^−^), base excess (BE), sodium (Na^+^), potassium (K^+^), and calcium (Ca^2+^) as previously described (Charest-Pekeski et al., 2021; Darby et al., 2021). In a subset of fetuses (*n* = 21, 105 ± 7 days GA), the carotid artery (CA) was instrumented and fetal BP, and HR were measured and continuously recorded in LabChart 8 Pro (ADInstruments Inc., Colorado Springs, United States) (Charest-Pekeski et al., 2021). Following *in utero* experiments gilts and their fetal pigs were humanely euthanized with an intravenous overdose of sodium pentobarbital (Virbac, New South Wales, Australia).

### Surgical Protocol for Pumped AP Studies

Pregnant Yucatan pigs (*n* = 19; 101 ± 4 days GA; term = 115 days) were anaesthetized with an I.M. injection of 10 mg/kg ketamine hydrochloride, 0.20 mg/kg acepromazine, and 0.015 mg/kg atropine sulfate (CDMV Inc., Saint-Hyacinthe, Canada), with maintenance of general anaesthesia with inhalation of 2–3% isoflurane (Fresenius Kabi Canada, Toronto, Canada). To prevent aortocaval compression, anaesthetized sows were positioned on the operating table in the left lateral position. Umbilical blood flow was measured *in utero* in four Yucatan sows in Toronto using cine phase contrast MRI as described in our previous publication (Charest-Pekeski et al., 2021). A lower antero-lateral laparotomy was performed for a caesarean section. Following the delivery of all fetal pigs, sows were humanely euthanized with 106 mg/kg Euthanyl (CDMV Inc., Saint-Hyacinthe, QC, Canada).

### Fetal Surgical Procedures

In a subset of fetuses (*n* = 11/13 successful pumped AP experiments), a small incision was made along the right side of the neck to expose the jugular vein (JV) and CA (Darby et al., 2021; Schrauben et al., 2021). A size-matched custom-made PVC tubing was then inserted in the JV and CA for monitoring of central venous pressure (CVP) and fetal mean arterial pressure (MAP), respectively. The tubing was stabilized using silk sutures, and the neck incision sutured closed. Fetuses were delivered to minimize excessive torsion and stretching of the umbilical cord to reduce the greater risk of vasospasm than in sheep and were positioned on the maternal abdomen and subsequently weighed. Fetal pigs were then anaesthetized using an I.M. injection of 1 mg/kg rocuronium bromide (Sandoz Inc., Mississauga, ON, Canada), and 5 mg/kg ketamine hydrochloride (CDMV Inc., Saint-Hyacinthe, QC, Canada). Fetal normothermia was maintained by continuously bathing the fetus and the umbilical cord in warmed normal saline. The umbilical cord was treated with a topical application of 100 mg/kg papaverine hydrochloride (Sandoz Inc., Boucherville, QC, Canada).

In all AP studies, umbilical catheters were then placed in both UAs and the UV (12-gauge and 10-gauge custom-made cannulas, respectively), and secured into place using silk sutures (Ethicon Inc., New Jersey, United States) at the insertion of the cannula within the vessel. In smaller fetuses, UA cannulas were downsized to 14 GA angiocaths (Becton Dickinson Canada Inc., Mississauga, ON, Canada). Cannulas were then connected to the AP circuit described below, *via* modified ¼″ perfusion adapters (Medtronic of Canada Ltd., Brampton, Canada) to facilitate the initiation of UV flows. Re-positioning of the umbilical catheters was performed as needed to assist in the establishment of flow.

### AP Circuit Design

**FIGURE 10:**
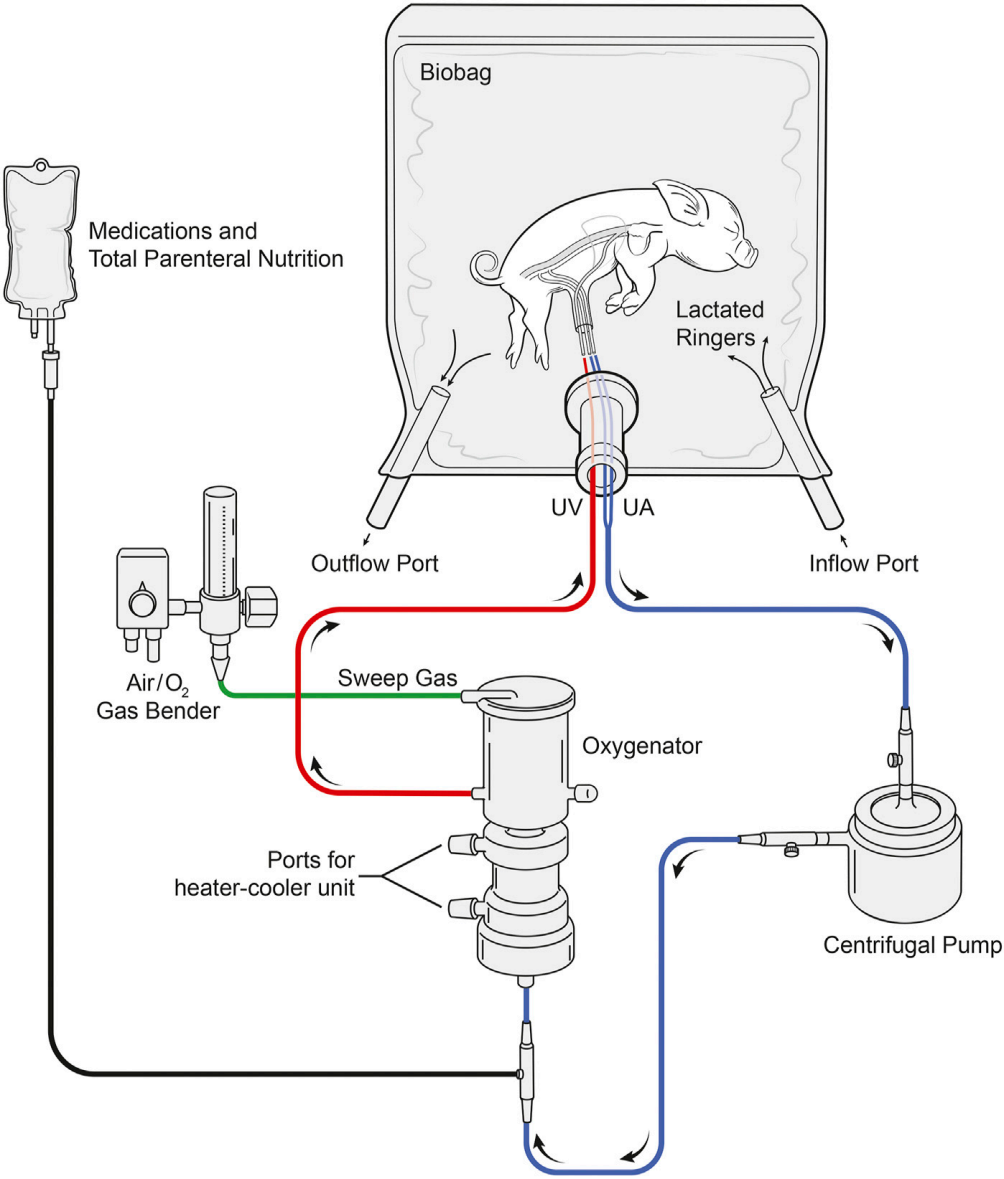
Layout of the pumped AP circuit. Fetal pigs (*n* = 13) were delivered via caesarean section, cannulated via the UA’s and UV, and maintained using a pumped AP circuit. Fetal pigs (*n* = 12) were also maintained using the same circuit design but without a centrifugal pump. Blood passed through the UAs and the centrifugal pump before entering the inflow port of the oxygenator. Oxygenated blood then passes through the oxygenator outflow port before returning to the heart via the UV. Sweep gas supplied the oxygenator with medical air and oxygen and the Biobag was filled with warmed LR solution to maintain fetal normothermia.

The arteriovenous AP circuit consisted of a prototype centrifugal pump (Chalice Medical Ltd., Worksop, England), connected to a low-resistance membrane oxygenator, and modified commercially available intravenous umbilical arterial and venous cannulas (length: ½″-1″) *via* 1/4″ internal diameter (ID) x 1/16″ (WT) wall thickness, 3/16″ ID x 1/16″ WT TYGON PVC tubing with P.h.i.s.i.o coating and cut to 8″-20″ lengths (LivaNova PLC., London, England). In two experiments, the Quadrox-I neonatal bypass oxygenator was used (Maquet Cardiopulmonary, Rastatt, Germany; Table 2); however, this was subsequently changed to the commercially available Chalice Paragon neonatal poly-methyl pentene ECMO oxygenator (Chalice Medical Ltd., Worksop, England; *n* = 11; Table 2). Total priming volumes for these neonatal membrane oxygenators were 40 and 65 ml, respectively. Circuits were primed with plasmalyte (Baxter Inc., Mississauga, Canada) and then replaced with heparinized maternal blood, which was continuously recirculated to prevent clotting within the circuit and warm the blood prior to connection to the fetus. UA blood passed through the oxygenator inlet, exiting through the outlet before returning to the heart *via* the UV (Figure 10). The sweep gas supplied the oxygenator with a mixture of medical air and oxygen (FiO_2_ range: 21%–40%) and was titrated to achieve normal physiologic levels of UV PO_2_ of 35–40 mmHg and a PCO_2_ of 50–60 mmHg (Acharya et al., 2016) based on *in utero* fetal pig data.

### Fluid Incubation

Following cannulation, fetal pigs were enclosed in a custom-made silicone infused thermoplastic polyurethane film “biobag”. Approximately 2–4 L of lactated ringer (LR; Baxter Inc., Mississauga, ON, Canada) crystalloid solution was warmed to 39°C ± 1°C in a large fluid reservoir and circulated *via* the inflow and outflow ports of the Biobag every 10 h. The Biobag was equipped with a temperature port that facilitated monitoring of fluid temperature using a temperature probe (ADInstruments Inc., Colorado Springs, CO, United States). The biobag was covered to prevent transmission of light to the fetus to better simulate uterine conditions and maintained at an appropriate temperature using a contact heat pad underneath the biobag and an overhead heater.

### Fetal Pig Maintenance on the AP Circuit

Following cannulation and transition to the AP, fetal pigs received a maintenance infusion of 6 µg/kg/h prostaglandin E1 (Pfizer Canada Inc., Kirkland, Canada) with 100 units/kg/h heparin (Fresenius Kabi Canada, Toronto, ON, Canada) to maintain patency of the ductus arteriosus, and prevent clotting in the AP circuit (Usuda et al., 2019). Ductal patency was confirmed by echocardiography throughout the duration of support for animals maintained on both AP circuits (Figures 4A,B and Supplementary Video S1)*.* Dextrose (418 mg/kg/h; Pfizer Canada Inc., Kirkland, Canada) was delivered *via* the circuit for the first 8 h of AP support, which was then exchanged for a neonatal total parenteral nutrition solution composed of amino acids (25 g/L), dextrose (100 g/L), Na^+^ (25 mmol/L), K^+^ (20 mmol/L), chloride (24 mmol/L), Ca^2+^ (12 mmol/L), phosphorus (12 mmol/L), magnesium (3 mmol/L), acetate (8 mmol/L), zinc (46 µmol/L), and copper (6.3 µmol/L) with the goal of supporting fetal energy requirements and maintaining fetal glucose concentrations of ≥5.6 mmol/L. Calcium chloride (80 mg/kg; Omega Laboratories Ltd., Montreal, Canada) and heparin (100 units/kg) were dosed empirically to achieve a target Ca^++^ concentration of ≥1.4 mmol/L, and an activated clotting time of 250–300 s, respectively. In early experiments (*n* = 7/13 successful experiments), 6 mg/kg/h of papaverine was administered I.V. to prevent vasospasm of the umbilical vessels and augment circuit flows. In a subset of experiments (*n* = 3/13), this was subsequently changed to a maintenance infusion of 30 µg/kg/h milrinone lactate (Aurobindo Pharma, Hyderabad, India). Reconstituted hydrocortisone (8 mg/kg/day, Pfizer Canada Inc., Kirkland, Canada) and a broad-spectrum empirical antibiotic (piperacillin/tazobactam; 300 mg/kg/day, Sandoz Inc., Boucherville, QC, Canada) was administered I.V. every 6 and 8 h, respectively. Whole maternal blood was transfused (10 ml/kg) to replete circulating blood volume following circuit phlebotomy. In addition, rocuronium bromide (1 mg/kg) and ketamine hydrochloride (5 mg/kg) were given for excessive fetal movements or perceived fetal agitation. Albumin (25%; CSL Behring Canada Inc., Ottawa, Canada) and furosemide (0.5 mg/kg; Pfizer Canada Inc., Kirkland, Canada) were given intermittently during longer experiments to address perivascular edema and hydrops fetalis.

Fetal CA, and umbilical arterial and venous blood gases including PO_2_, PCO_2_, pH, Hb, HCO_3_^−^, SO_2_, BE, Na^+^, K^+^, lactate, glucose and ACT were sampled every 1–3 h and analyzed using a handheld blood analyzer (Abbott Point of Care Inc., Nepean, Canada). Fetal oxygen delivery (DO_2_) and consumption (VO_2_) were calculated based on the combination of UV and UA oxygen carrying and indexed UV flow indexed to fetal weight (measured at surgery).

$$DO_{2}=Indexed\;UV\;flow*\left(1.36*\left[Hb\right]*UV\;SO_{2}\right)\;$$

$$VO_{2}=Indexed\;UV\;flow*\left(UV\;SO_{2}-UA\;SO_{2}\right)*1.36*\left[Hb\right]$$

$$Oxygen\;extraction\;fraction\;\left(\%\right)=\left(\left(\left(\text{UV SO}2-\text{UA}\;{\text{SO}}_{2}\right)\right)\text{/}\left(\mathrm{UV}\;SO_{2}\right)\right)*100$$

### Physiologic Monitoring

UV flow was continuously measured using a HXL 3/16″ tubing flow probe (Transonic) and CVP, fetal BP, pre-pump circuit pressure, pre-oxygenator circuit pressure, and post-oxygenator circuit pressure were measured using Deltran fluid filled blood pressure transducers (ADInstruments Inc., Colorado Springs, CO, United States). Data were sampled at 1000 Hz, digitized, and continuously recorded using LabChart Pro 8 (ADInstruments Inc., Colorado Springs, CO, United States). At the end of each study, the data was extracted in consecutive 30-s intervals and analyzed in Excel (Microsoft Corporation, Washington, United States).

### Quantification of UV Blood Flow *in utero* Using MRI: 3-D Volumetry and Cine Phase-Contrast

*In utero* fetal weight and UV flow were measured in sixteen fetuses from four pregnant Yucatan pigs (*n* = 4; gestational age 107 ± 3 days GA) as previously described using a 3 T magnetic resonance imaging (MRI) system (Charest-Pekeski et al., 2021).

### Statistical Analysis

Comparisons of anthropometric data between pumpless and pumped AP circuits were analyzed using a two-way ANOVA with Bonferroni correction for multiple comparisons. Comparison of the duration of AP support between pumpless and pumped circuits was analyzed using a Mann-Whitney *U* test. Changes in temperature, HR, and UV flow between pumpless and pumped AP circuits over the first 3 h of support were analyzed using a mixed-effect model, with a Bonferroni correction for multiple comparisons. Differences in mean indexed and absolute UV flow and HR between *in utero*, pumpless and pumped AP circuits were compared using a repeated measures one-way ANOVA with a Bonferroni correction for multiple comparisons. Analysis of fetal HR and indexed UV flow were performed using a linear regression. Comparisons of mean blood gases, electrolytes, lactate, and glucose concentrations between animals studied *in utero*, or on pumpless and pumped AP circuits were analyzed using a repeated measure one-way ANOVA, and a Kruskal-Wallis test (when appropriate) with Bonferroni correction for multiple comparisons. UV flow, HR, fetal BP, CVP, and temperature are presented in 5-min averages. Time post-cannulation versus UV flow is presented in 1 h averages and analyzed using a one-way ANOVA with Bonferroni correction for multiple comparisons. Differences in MAP between fetal pigs studied *in utero* and those maintained using a pumped AP were analyzed using a mixed-effect model with Bonferroni correction for multiple comparisons. Indexed UV flow versus oxygen extraction was analysed using a linear regression. *p < 0.05 was considered statistically significant. All statistical analyses were performed using Prism 9 (GraphPad, San Diego, United States). Data are presented as mean ± standard deviation (SD), unless otherwise indicated.

## Data Availability

The data that support the findings of this study are available from the corresponding author upon reasonable request.
